# Myoepithelial Carcinoma of Sinonasal Cavity: Peculiar Diagnosis, Conventional Treatment

**DOI:** 10.1007/s12070-023-03989-7

**Published:** 2023-06-19

**Authors:** Diksha Dinker, Keshava Rajan, Swati Sharma, Naveena AN Kumar

**Affiliations:** 1 Department of Surgical Oncology, Manipal Comprehensive Cancer Care Centre, Kasturba Medical College, Manipal Academy of higher Education (MAHE), Manipal, 576104 Karnataka India; 2 Department of Pathology, Kasturba Medical College, Manipal Academy of Higher Education, 576104, Manipal, Karnataka India

## Abstract

Myoepithelial carcinoma is a morphologically diverse tumor which either arises de novo or from the malignant transformation of its benign counterpart i.e. myoepithelioma. These are relatively lesser known entities and are rarely found in head and neck region. Although rare, their first presentation is usually a painless growing mass as seen in our case presentation as well and are infamous for lymph node recurrence and distant metastasis. Due to their clinical presentation and varied morphology these become tedious to diagnose and pose difficulty for a surgeon when presented at a later date due to their effect on the adjacent vital structures. We report a case of myoepithelial carcinoma in head and neck region arising from the nasal cavity, it’s mass effect on the adjacent vital organs and the diagnosis and treatment plan to render the patient free of this tumor, preservation of the vision and keeping the recurrence of the tumor at bay.

## Introduction

Myoepithelial carcinoma (MC) is a malignant salivary gland neoplasm and a relatively rare entity as it represents about 0.4–0.6% of all salivary gland tumors and 1.2–1.5% of carcinomas [1]. Its most common occurrence is noted in parotid gland but some studies have suggested a higher predilection in minor salivary glands in palate [2]. These occur de novo or as a malignant transformation of their benign counterparts known as myoepithelioma. Metastasis is generally seen in regional lymph nodes and distant sites like lungs, kidney, brain and bones [3]. Hence, their propensity of distant metastasis and loco regional recurrence makes it necessary to diagnose them early, intervene appropriately.

## Case Presentation

A middle-aged gentleman presented to us with the complaint of blocked nose on the right associated with bleeding for three months. He also reported to have blurred vision since a month. The patient didn’t have any comorbidities and claimed to have no habit history. Upon arrival, patient was thoroughly evaluated clinically. On inspection, there was no visible abnormality locally in the facial region or the neck.

**Fig. 1:**
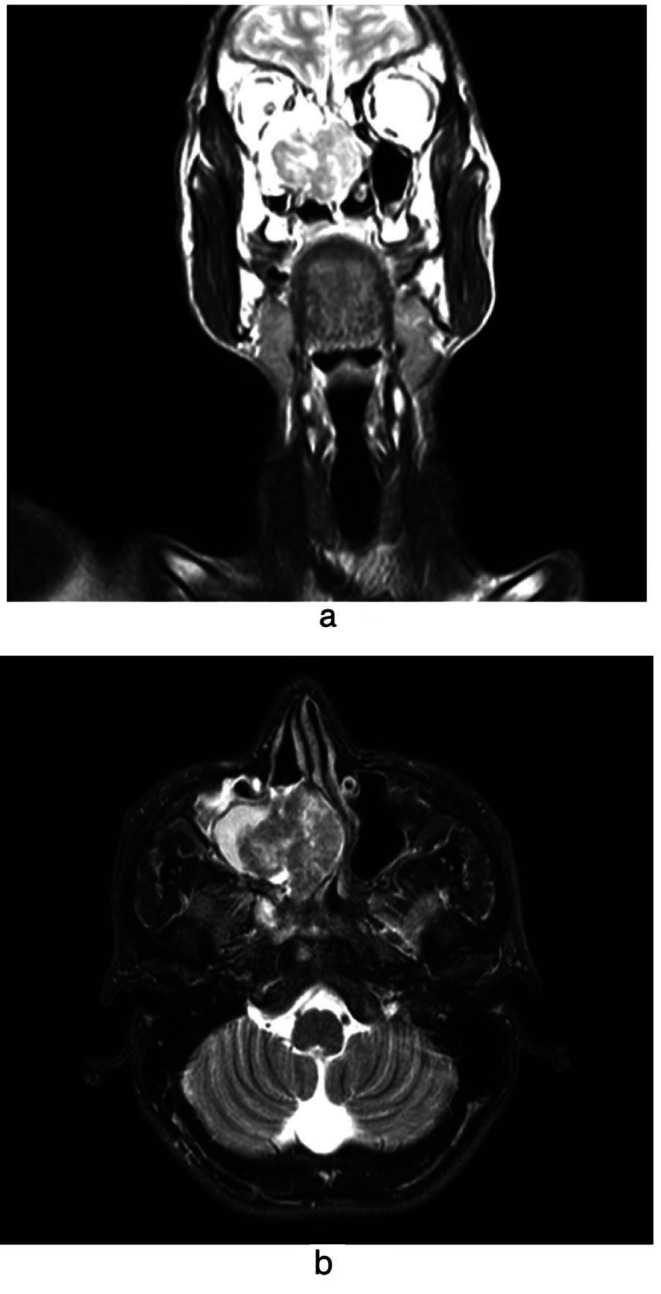
**1a**: MRI showing lesion in coronal section. **1b**: MRI showing lesion in axial section

**Fig. 2:**
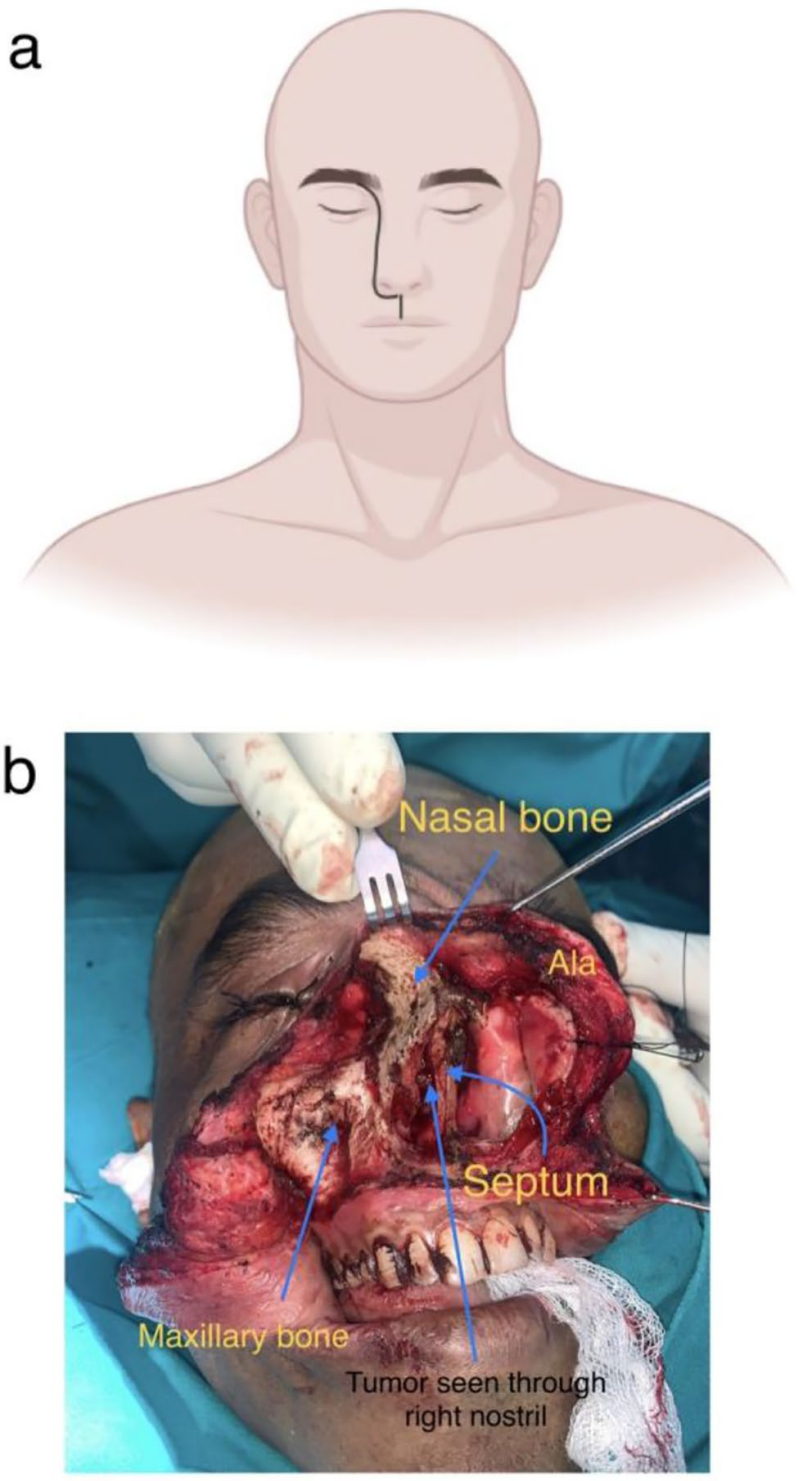
**2a**: Weber Ferguson with Lynch extension. **2b**: Presentation of tumor on exposure

**Fig. 3:**
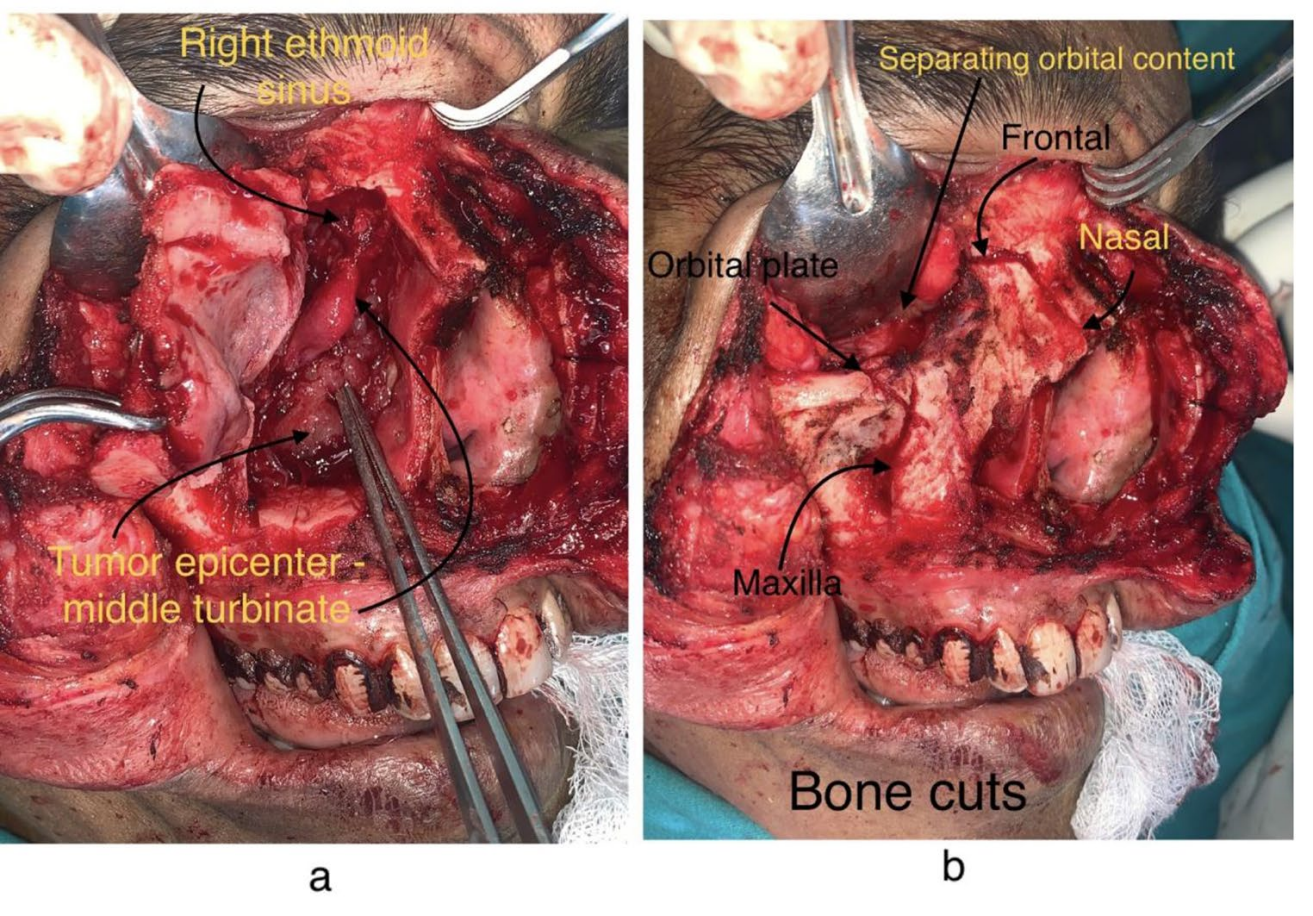
**3a, 3b**: Tumor presentation on bone cuts and debulking

**Fig. 4:**
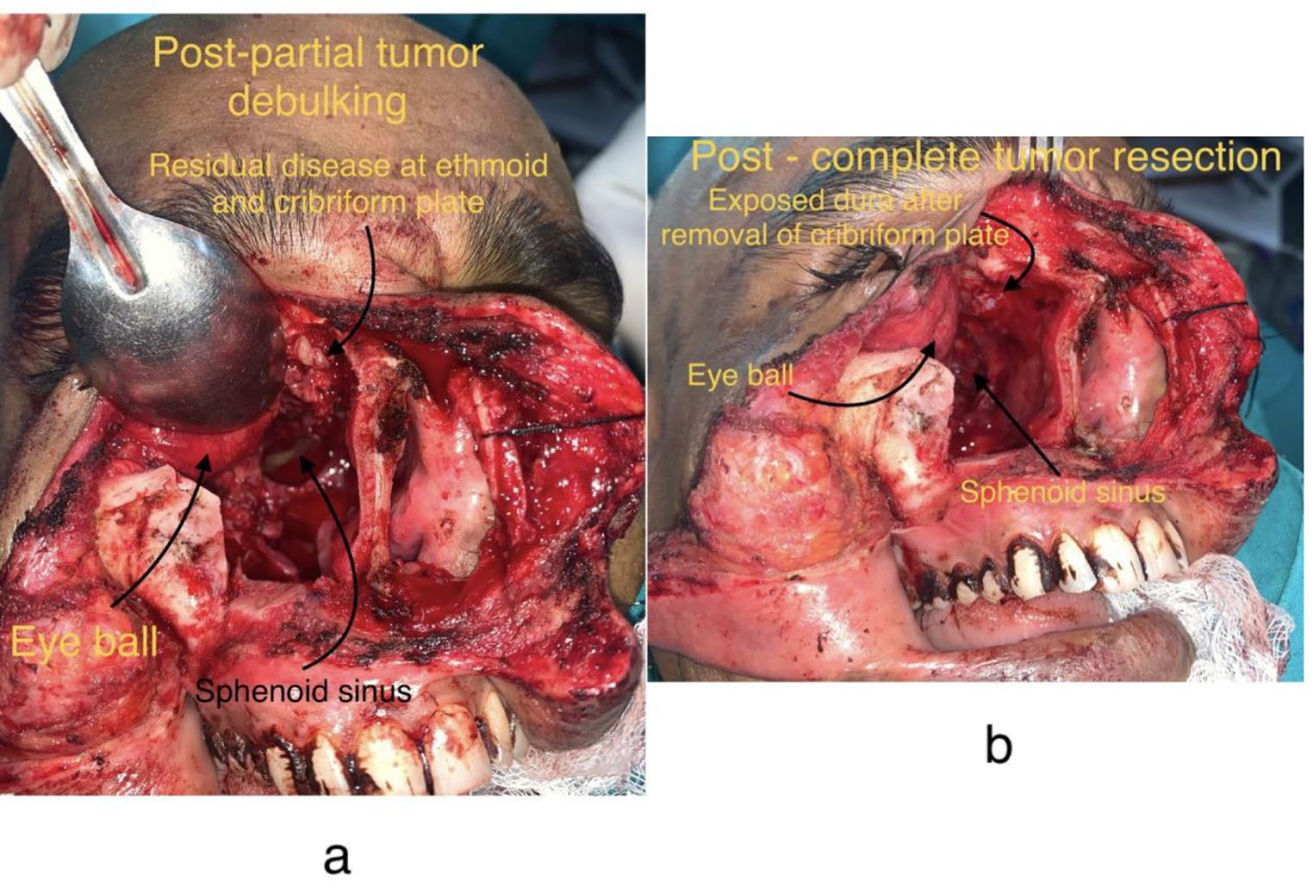
**4a, 4b**: Post partial tumor debulking

On palpation, tenderness was evident with respect to the right paranasal region of the face and the transillumination test turned out to be positive for the same region. No other visible swelling was found on further nasal examination with a speculum. This required imaging to evaluate for the true extension of disease.

Contrast enhanced computed tomography (CT) was chosen to evaluate for the extensions of the lesion which displayed an irregular growth involving the right sinonasal cavity extending up to medial wall of orbit, inferior orbital fissure and sphenoid sinus. MRI revealed heterogeneously enhancing lesion epicentered in right nasal cavity involving inferior and middle turbinates. Laterally involving the medial and inferior wall of the right orbit without displaying any involvement of the eyeball. There was extraconal extension causing mass effect on inferior & medial rectus of right orbit. Inferiorly the lesion extended in ethmoid sinus and eventually till cribriform plate. There was no intracranial extension. Further inferiorly it appeared to be abutting hard palate without any obvious evidence of oro antral communication. Although cavernous sinus & Internal carotid artery appeared spared (Fig. 1a and b).

**Fig. 5:**
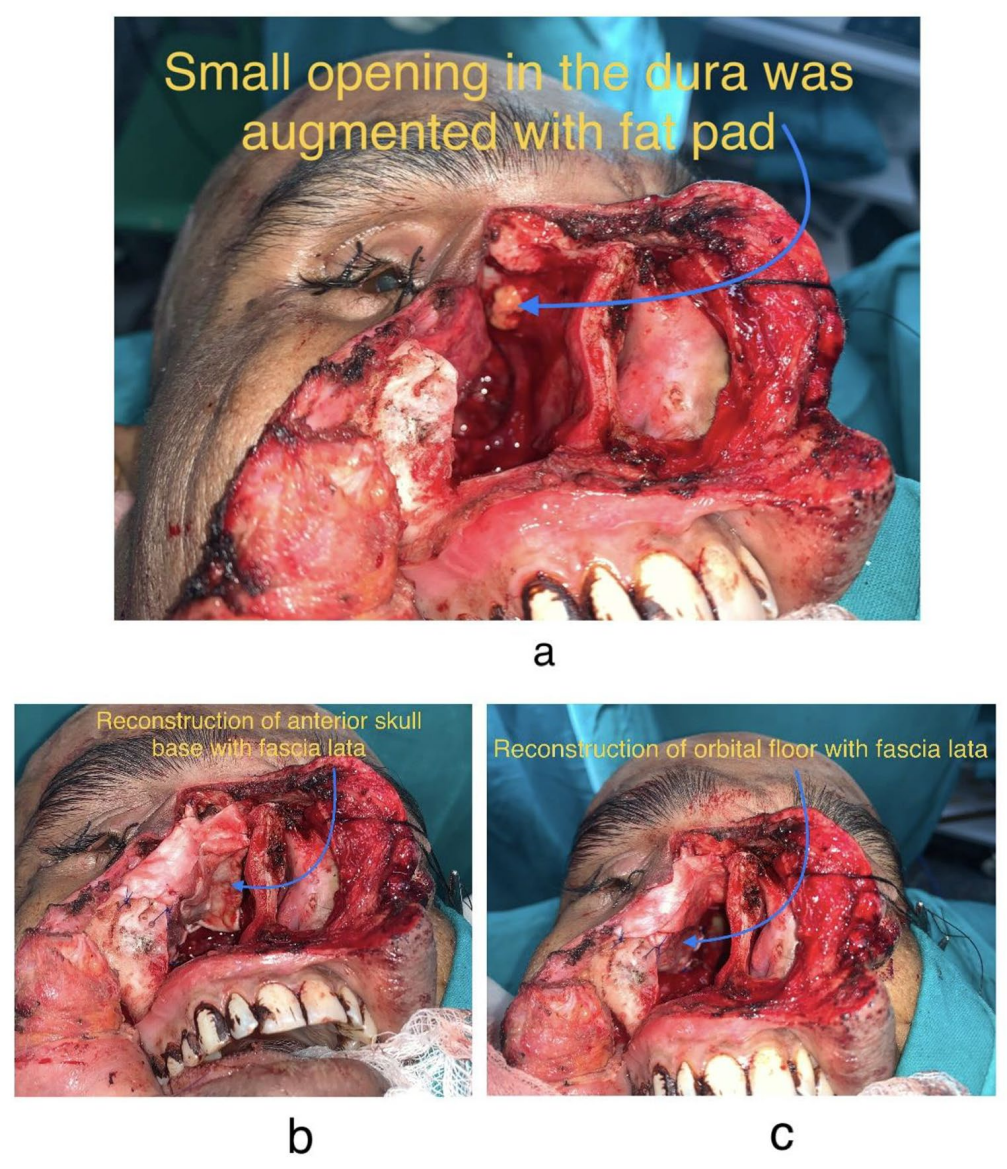
**5a, 5b, 5c**: Reconstruction with Tensor fascia lata and repair of dural tear with Buccal fat pad

**Fig. 6:**
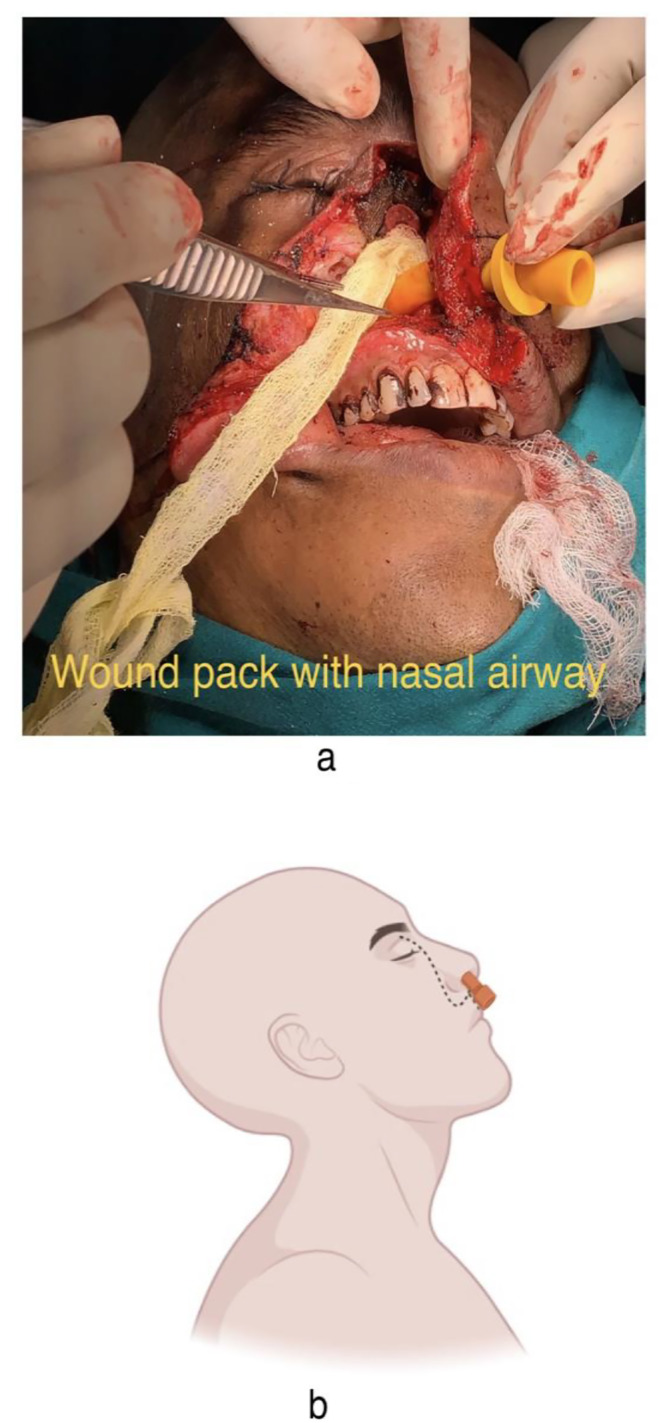
**6a, 6b**: Placement of BIPP pack & Nasopharyngeal airway

The case was discussed in the multidisciplinary tumor board and was decided for upfront surgery. As per the extensive nature of the lesion, a surgical approach was decided on in order to relieve the pressure effects of the lesion on the surrounding vital structures. Alongside achieving appropriate margins, in toto removal of the lesion and a R0 resection were a few goals kept in mind in order to achieve complete clearance of the tumor.

Weber Ferguson incision with Lynch extension was advocated to expose the lesion. Lateral nasal wall was exposed completely exposing the infraorbital rim till its lateral extent. Orbital floor was explored and tumor was noted to be infiltrating the floor and the posterior extent of tumor was noted. Upon adequate visualization, Orbital fat pad was noted to be free from tumor. (Figure 2a and b)

**Fig. 7:**
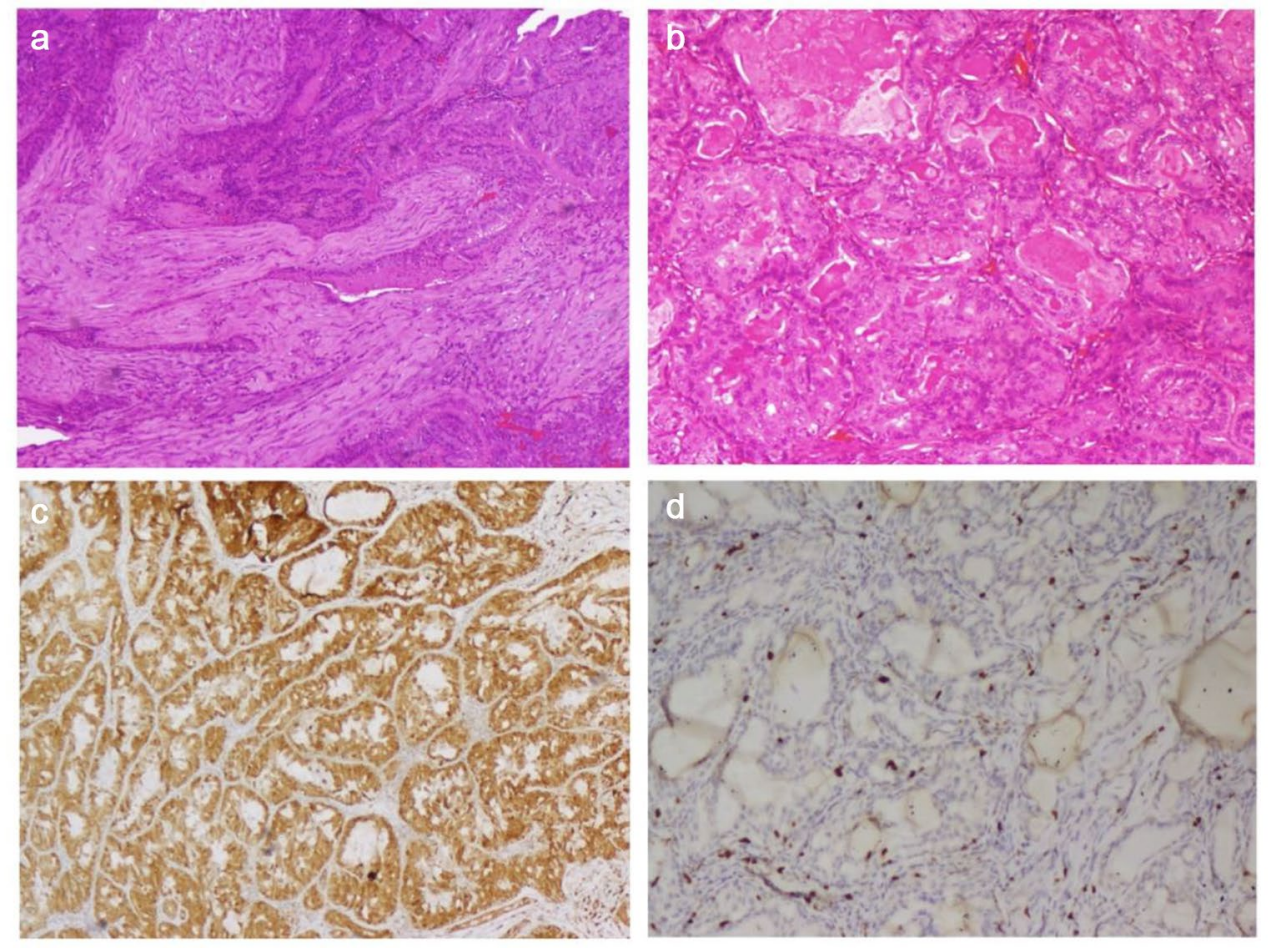
**7a, 7b**: Histopathologic section of tumor showing infiltrating tumor composed of cells arranged in glandular and branching tubular pattern ( H & E, 50X & H& E, 100X). Fig **7c, 7d**: Immunohistochemistry staining positive for S100, Ki- 67 (S-100, 50X & Ki-67, 50X)

Lamina papyracea was exposed as continuing the dissection further, maxillary bony wall was exposed to visualize the sinus and tumor was seen extending in right nasal cavity without any obvious extension in the left nasal cavity (Fig. 3a and b). Septal wall was noted to be free & hence the tumor was excised in toto followed by piece meal excision near the ethmoid sinus and cribriform plate. The tumor lining and sinus mucosa were excised around the sphenoid sinus and no tumor residual was left hence achieving R0 resection which was the one of the most important goals of the treatment plan.

Eyeball was preserved and temporary tarsorrhaphy was done as well (Fig. 4a and b). Partial floor defect created by the resection was reconstructed by Tensor Fascia Lata harvested from patient’s right thigh. It was used for creating a sling ligament for supporting orbital contents. A smaller piece of the same graft was used along with Buccal Fat Pad for closing a minute dural tear at the time of resection in the anterior aspect (Fig. 5a, b and c). No cerebrospinal fluid (CSF) leak was noticed at the time of surgery. Right nasal cavity was packed with Bismuth Iodoform Paraffin Paste (BIPP) impregnated ribbon gauze and Nasopharyngeal airway was placed thereafter in the right nasal cavity. Wound then closed in layers (Fig. 6a and b). Patient furthermore continued with adequate antibiotic cover post op and had an uneventful recovery and was eventually discharged after 5 days in a stable condition.

Histopathology report from a section of tumor showed an infiltrating tumor composed of cells arranged in glandular and branching tubular pattern. These glands were lined by bilayered epithelium. The luminal cells had pleomorphic nucleoli, moderate cytoplasm, inspissated eosinophilic secretions. Oncocytic cell change was noted with basement membrane like hyalinized materials. The section also displayed foci of high-grade transformation. Immunohistochemistry (IHC) reported positive CK7, SMA, P40, GFAP, S100, CD117 (1+) markers and negativity for CK20 and AR. Ki67% value was noted to be less than 10% (Fig. 7). All resection margins were negative.

On first follow-up, the patient presented with mild diplopia, which was normalized with conservative management. Vision was preserved and improved without any blurring. No signs of epiphora or rhinorrhea were noted. Adjuvant radiotherapy to the surgical bed and the neck was planned as per the multidisciplinary tumor board decision. No clinical and radiological signs of locoregional recurrence at 12 months of follow up.

## Discussion

Myoepithelial carcinoma is a rare salivary gland tumor which can also exhibit in skin and other soft tissues. The reported incidence of this entity is 0.2% of all salivary gland tumors [1]. As the name suggests it’s composed of a mixture of two elements namely *myo* which means muscle and epithelium. Pertaining to it’s terminology, it shows dual characteristics of both the phenotypes just like it’s benign counterpart i.e. myoepthelioma with an inclusion of malignancy.

These tumors have a female predilection [4] and commonly present in the sixth decade of life [1, 5–7] It also shows propensity for high-risk features such as peri neural invasion, lympho-vascular infiltration, loco regional recurrence and is notorious for distant metastasis [8].

Literature reports this as a lesser encountered entity but in fact a lack of recognition of its diversity and diagnosis might be the reason behind a relatively small number of reported cases.

Stromeyer was the first one to describe this rare entity in 1975 [9] and Barnes et al. went on to review three cases of myoepithelial carcinoma in head & neck in 1985 [10] but it was Dardick who eventually gave a description that was crucial in increasing awareness of its nature, presentation and diagnosis.^[[11][12]]^ Most reported cases have found out that 75% of myoepithelial carcinomas arise in the major salivary glands viz. parotid followed by minor salivary glands [6, 9, 13] On the contrary, a review of 51 such cases at TMH by *Shubhada et al.* showed predilection for minor salivary glands present in palate (n = 15), buccal mucosa (n = 7), nasal cavity (n = 5), maxilla(n = 3), lower alveolus (n = 3), tongue (n = 2) and floor of mouth (n = 1) [2].

Since it’s an entity that can also arise from minor salivary glands, the usual histopathological examination doesn’t prove to be useful in determining the depth of invasion hence IHC (Immuno histo chemistry) plays an important role in diagnosis of this tumor. Specific markers like vimentin, cytokeratin (CK), epithelial membrane antigen (EMA), CD 10, smooth muscle actin (SMA), S 100 protein, p63 and Calponin show positivity and there’s carcinoembryonic antigen (CEA) negativity on analysis [2]. Histopathologically, this tumor shows wide morphological variation in the form of epitheliod type which is the most predominant cell type followed by plasmacytoid, spindle, clear, stellate and finally a mixed cell pattern. A heterogenous pattern showing nuclear atypia predisposes it to it’s aggressive behaviour. Another point to be noted here is the rarity of this lesion in paranasal sinuses in head and neck region. As these generally arise from major salivary glands namely parotid gland, their occurrence in minor salivary glands is rare but not unknown.

From a surgical perspective, this entity poses difficulty in terms of its bulky nature as it mainly presents as a painless growing mass with or without numbness in facial region and reconstruction of the defect caused by its resection. As the tumor gets bulkier, it becomes more tedious to achieve the the goal of R0 resection. An incomplete removal of such a tumor can lead to recurrence and distant metastasis eventually. Seethala et al. reported positive margins, angiolymphatic invasion, necrosis, and myoepithelial anaplasia as predictors of decreased disease-free survival. In their population of 45 patients with follow-up data, 5- and 10-year disease-specific survivals were 93.5% and 81.8%, respectively; only three patients died of disease, but all had positive margins, angiolymphatic invasion, and necrosis on initial resection, with the eventual development of either regional or distant metastases [14]. Vazquez et al. in their Study of 246 patients with salivary gland myoepithelial carcinoma reported an overall 5-year disease-specific survival of 91.3%, with distant metastasis observed in only 4.5% of patients. A tumor size of more than 4 cm and high-grade histology (6.5% of patients) were found to be associated with increased mortality. Their treatment consisted of surgery, with adjuvant radiation administered to 39% of patients, although no survival benefit was found with the addition of radiation therapy [15].

Even in early lesions, unusually high-grade histology with significant nuclear atypia, necrosis, and elevated mitotic count as seen in our case report can lead to recurrence as depicted by *Park et al.* in their report [16]. As per a similar report by *Shubhada et al.*, there were 5 reported cases which arose from the nasal cavity even though there’s no description of the size of the lesion and whether or not these were removed surgically, a brief mention of the same reported recurrence in 2 out of 5 cases. Along with the site, there was also a high incidence of recurrence in cell types stellate, clear and spindle shaped variants. There has been a parallelism drawn between the size and the recurrence as well. In terms of follow up of minimum 24 months, available for 18 patients, 12 showed recurrence, 3 showed lymphatic metastasis, and 2 showed distant metastasis. The mean disease-free survival time calculated was 31.9 months [2].

Treatment approach for sinonasal epithelial myoepithelial carcinoma (EMC) is mainly a combination of open or endoscopic resection depending on the size of the lesion with or without adjuvant radiotherapy. The goal of surgery for EMC is margin-negative resection, with avoidance of major morbidity. The role for adjuvant radiation in salivary EMC is not established, although it may be recommended for patients with high-risk clinical or histologic features. An incomplete surgery can be predictive of recurrence.

Hence, even though these tumors remain diverse macro and microscopically, their diagnosis is the key to fabricate an appropriate treatment plan and the prediction of the prognosis depending on their presentation histopathologically.

## Conclusion

Myoepithelial carcinoma in head and neck is a rare entity. The proper histopathological diagnosis, multidisciplinary treatment approaches, well planned surgery to achieve R0 resection and adjuvant radiotherapy are the key components of treatment.
